# Quantitative firing pattern phenotyping of hippocampal neuron types

**DOI:** 10.1038/s41598-019-52611-w

**Published:** 2019-11-29

**Authors:** Alexander O. Komendantov, Siva Venkadesh, Christopher L. Rees, Diek W. Wheeler, David J. Hamilton, Giorgio A. Ascoli

**Affiliations:** Krasnow Institute for Advanced Study, George Mason University, 4400 University Drive, MS 2A1, Fairfax, Virginia 2230 USA

**Keywords:** Data publication and archiving, Action potential generation

## Abstract

Systematically organizing the anatomical, molecular, and physiological properties of cortical neurons is important for understanding their computational functions. Hippocampome.org defines 122 neuron types in the rodent hippocampal formation based on their somatic, axonal, and dendritic locations, putative excitatory/inhibitory outputs, molecular marker expression, and biophysical properties. We augmented the electrophysiological data of this knowledge base by collecting, quantifying, and analyzing the firing responses to depolarizing current injections for every hippocampal neuron type from published experiments. We designed and implemented objective protocols to classify firing patterns based on 5 transients (delay, adapting spiking, rapidly adapting spiking, transient stuttering, and transient slow-wave bursting) and 4 steady states (non-adapting spiking, persistent stuttering, persistent slow-wave bursting, and silence). This automated approach revealed 9 unique (plus one spurious) families of firing pattern phenotypes while distinguishing potential new neuronal subtypes. Novel statistical associations emerged between firing responses and other electrophysiological properties, morphological features, and molecular marker expression. The firing pattern parameters, experimental conditions, spike times, references to the original empirical evidences, and analysis scripts are released open-source through Hippocampome.org for all neuron types, greatly enhancing the existing search and browse capabilities. This information, collated online in human- and machine-accessible form, will help design and interpret both experiments and model simulations.

## Introduction

Quantitative characterization of neurons is essential for understanding the functions of neuronal networks at different hierarchical levels. The hippocampus provides an excellent test-bed for this exploration as it is one of the most intensively studied parts of the mammalian brain, and is involved in critical functions including learning^1,2^, memory^3–5^, spatial navigation^6,7^, and emotional associations^8^.

Transmission of information between neurons is carried out by sequences of spikes, and firing rates are commonly believed to represent the intensity of input stimuli. Since the first discovery in sensory neurons^9^, this principle was generalized and extended to neurons from different brain regions, including the hippocampus^10^. However, it was also found that the firing rate of certain neurons is not constant over time, even if the stimulus is permanently applied. One form of such time-dependent responses is spike frequency adaptation, manifested in a decrease of firing rate^9^. Neurons can produce diverse firing patterns in response to similar stimuli due to the inhomogeneity in their intrinsic properties^11^. Both firing rates and temporal firing patterns have long been recognized to play important roles in neural information coding^12^.

In electrophysiological experiments *in vitro*, hippocampal neurons demonstrate a vast diversity of firing patterns in response to depolarizing current injections. These patterns are referred to by many names, including delayed, adapting, accommodating, interrupted spiking, stuttering, and bursting^13–19^. Uncertainties and ambiguities in classification and naming of neuronal firing patterns are similar to other widely spread terminological inconsistencies in the neuroscience literature, posing obstacles to effective communication within and across fields^20^.

Recent efforts aimed to classify firing patterns for identifying distinct electrical types of cortical neurons^21–24^. Notably, statistical analysis of a large set of electrical features of neocortical interneurons with different firing patterns from a single lab yielded a refinement of the physiological component of the Petilla Nomenclature^24^. However, comparisons across labs and experimental studies are typically limited to qualitative assessments of the illustrated firing traces or subjectively intuitive criteria. Moreover, firing pattern data are seldom unambiguously linked to neuron types independently defined by morphological and molecular criteria.

The Hippocampome.org knowledge base defines neuron types based on the locations of their axons, dendrites, and somata across 26 parcels of the rodent hippocampal formation, putative excitatory/inhibitory output, synaptic selectivity, and major and aligned differences in molecular marker expressions and biophysical properties^25^. Version 1.3 of Hippocampome.org identifies 122 neuron types in 6 major areas: 18 in dentate gyrus (DG), 25 in CA3, 5 in CA2, 40 in CA1, 3 in subiculum (SUB), and 31 in entorhinal cortex (EC). The core assumption of this identification scheme is that neurons with qualitatively different axonal or dendritic patterns, or with multiple substantial differences in other dimensions, belong to different types. For the majority of neuron types, Hippocampome.org reports 10 basic biophysical parameters that numerically characterize passive and spike properties (hippocampome.org/ephys-defs), consistent with other literature-based neuroinformatics efforts^26^.

Here, we developed an objective numerical protocol to automatically classify published electrophysiological recordings of somatic spiking activity for morphologically identified hippocampal neurons from Hippocampome.org. This process revealed specific firing-pattern phenotypes, potential neuronal subtypes, and statistical associations between firing responses and other properties. Inclusion of the classified firing patterns and their quantitative parameters, along with a comprehensive tabulation of the underlying experimental conditions, substantially extends the online search and browse functionalities of Hippocampome.org, providing a wealth of annotated data for quantitative analysis and modeling.

## Methods

### Data collection, extraction and digitization

The firing patterns of hippocampal neurons were classified based on their spiking responses to supra-threshold step-current pulses of different amplitude and duration as reported in peer-reviewed publications. Firing pattern parameters were extracted from electronic figures using Plot Digitizer (plotdigitizer.sourceforge.net) for all Hippocampome.org neuron types^25^ for which they were available (90 out of 122). A total of 247 traces were analyzed. We extracted values of first spike latency (i.e. delay), inter-spike intervals (ISIs), and post-firing silence (in ms), as well as slow-wave amplitude (in mV) for burst firing recording. For firing pattern identification and analysis, ISIs in each recording were normalized to the shortest inter-spike interval (*ISI*_*min*_) within that time series, to allow meaningful comparison.

All analyzed recordings were obtained in normal artificial cerebrospinal fluids (ACSF) from rodents (rats 85%, mice 12%, and guinea pigs 3%) generally described as “young adults” (ages ranging from 11 to 70 days for rats and from 10 to 56 days for mice). All firing traces considered in this report were recorded in slice preparations; 74% of electrophysiological traces were obtained using whole-cell patch clamp and 26% intracellular recording with sharp microelectrodes. All experimental conditions and solution compositions were extracted and stored with every recording and are available at Hippocampome.org as specified in the “Web portal” section below. Representative examples of ACSF and of solutions for pipette filling are shown in Supplementary Tables S1 and S2, respectively.

### Firing pattern classification

**Figure 1:**
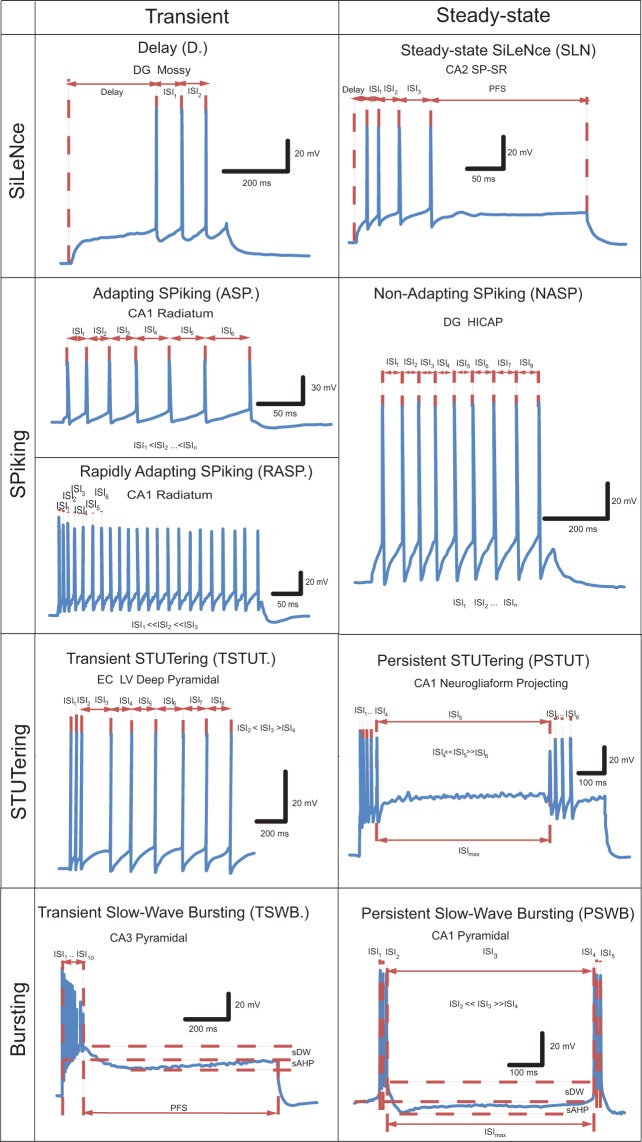
Firing pattern elements observable in hippocampal neurons *in vitro*. ISI - inter-spike interval, PFS – post firing silence, sDW – slow depolarization wave, sAHP – slow after-hyperpolarization. Original data extracted from Lübke *et al*.^16^ (D), Vida *et al*.^97^ (ASP), Pawelzik *et al*.^18^ (RASP), Hamam *et al*.^48^ (TSTUT), Chevaleyre and Seigelbaum^28^ (TSWB), Mercer *et al*.^98^ (SLN), Mott *et al*.^39^ (NASP), Fuentealba *et al*.^29^ (PSTUT), and Golomb *et al*.^31^ (PSWB, spontaneous bursting in Ca^2+^-free ACSF).

Hippocampal neuron types display a variety of firing pattern elements (FPE) in both their transient and steady state responses to continuous stimulation (Fig. 1). Specifically, transients (which we label by dot-notation) can be visually differentiated into delay (D.), adapting spiking (ASP.), rapidly adapting spiking (RASP.), transient stuttering (TSTUT.), and transient slow-wave bursting (TSWB.). Steady states include silence (SLN), non-adapting spiking (NASP), persistent stuttering (PSTUT), and persistent slow-wave bursting (PSWB).

In certain cases, a constant current injection elicits firing patterns consisting of single firing pattern elements (NASP, PSTUT or PSWB). In other cases, complex firing patterns are observed as sequences of two or more firing pattern elements, such as delayed non-adapting spiking (D.NASP), silence preceded by adapting spiking (ASP.SLN), and non-adapting spiking preceded by delayed transient slow-wave bursting (D.TSWB.NASP). Experimental recordings without identifiable steady states were deemed uncompleted firing patterns (e.g. ASP., D.ASP., or RASP.ASP.).

In order to define the firing pattern elements unambiguously, we developed a set of quantitative classification criteria (Table 1). The transient response was classified as delayed (D.) if the latency to the first spike was longer than the sum of the first two inter-spike intervals (*ISI*_1_ and *ISI*_2_). Similarly, post-firing silence (PFS) was considered to be a steady state (SLN) if it exceeded the sum of the last two inter-spike intervals (*ISI*_*n-*1_ and *ISI*_*n*_). In addition, post-firing silence had to last at least twice the longest inter-spike interval (*ISI*_*max*_).

**Table 1:** Principles of classification of firing pattern elements.

Firing pattern element		Transient responses	Steady-state responses	Characteristics of responses	Values of parameters
Silence		Delayed (D.)		${Delay} > {DF}\frac{{IS}{{I}}_{1}+{IS}{{I}}_{2}}{2}$	*DF* = 2
			SiLeNce (SLN)	$$\begin{array}{c}{PFS} > {SF}\frac{{IS}{{I}}_{{n}}+{IS}{{I}}_{{n}-1}}{2}\\ {PFS} > {SF}\ast {IS}{{I}}_{{\max }}\end{array}$$	*SF* = 2
Spiking		Adapting Spiking (ASP.)		*ISI*_*1*_ < *ISI*_*2*_ < *ISI*_*n*_; to compare 2 parameter fit *(Y* = *a*_*1*_*X* + *b*_*1*_) and 3 parameter fit *(Y* = *a*_1_*X* + *b*_1_*; Y* = *b*_2_)	*p*_*2*,1_ < 0.05 *p*_*3,2*_ > 0.025 *a*_1_ > 0.003
		Rapidly Adapting Spiking (RASP.)		$$\begin{array}{c}{IS}{{I}}_{1} < < {IS}{{I}}_{2} < < {IS}{{I}}_{3}\\ {Y}={{a}}_{1}{X}+{{b}}_{1}\\ {{a}}_{1} > {{S}}_{{RASP}}\end{array}$$	*S*_*RASP*_ = 0.2
			Non-Adapting Spiking (NASP)	*ISI*_*1*_ *≈ ISI*_2_ *… ≈ ISI*_*n*_*;*to compare 1 parameter fit *(Y* = *b*_1_) and 2 parameter fit *(Y* = *a*_1_*X* + *b*_1_)	*p*_*2*,1_ > 0.05
Interrupted	Stuttering	Transient STUTering (TSTUT.)		$$\begin{array}{c}{ISIi} > {Fpre}\ast {ISIi}-1\\ {IS}{{I}}_{{i}} > {{F}}_{{post}}\ast {IS}{{I}}_{{i}+1}\\ \frac{\mathop{\sum }\limits_{{j}={i}}^{{n}}{IS}{{I}}_{{j}}}{{n}-{j}} > {{F}}_{{pre}}\frac{\mathop{\sum }\limits_{{j}=1}^{{i}-1}{IS}{{I}}_{{j}}}{{j}}\\ \forall {j} < {i}-{1}:\frac{{1}}{{IS}{{I}}_{{j}}} > {{f}}_{{\min }}\end{array}$$_(T1.1)_	*F*_*pre*_ = 2.5 *F*_*post*_ = 1.5 *f*_*min*_ = 25 Hz *i* = 2,3,4
			Persistent STUTering (PSTUT)	$\frac{{IS}{{I}}_{{i}}^{{\max }}}{{IS}{{I}}_{{i}-1}}+\frac{{IS}{{I}}_{{i}}^{{\max }}}{{IS}{{I}}_{{i}+1}} > {{F}}_{{PSTUT}}$	*F*_*PSTUT*_ = 5
	Slow-Wave Bursting	Transient Slow-Wave Bursting (TSWB.)		Inequalities T1.1, *SWA* > *SWA*_*min*_	*F*_*pre*_ = 2.5 *F*_*post*_ = 1.5 *f*_*min*_ = 25 Hz *i* = 2,3,4 *SWA*_*min*_ = 5 mV
			Persistent Slow-Wave Bursting (PSWB)	$\frac{{IS}{{I}}_{{i}}^{{\max }}}{{IS}{{I}}_{i-1}}+\frac{{IS}{{I}}_{{i}}^{{\max }}}{{IS}{{I}}_{{i}+1}} > {{F}}_{{PSWB}}$ *SWA* > *SWA*_*min*_	*F*_*PSWB*_ = 5 *SWA*_min_ = 5 mV

**Abbreviations:** *a*_*1*_ – slope of linear fitting for normalized ISIs *vs* normalized time; *DF* – delay factor; *f*_*min*_ – minimum frequency of stuttering or bursting; *F*_*pre*_, *F*_*post*_, *F*_*PSTUT*_, *F*_*PSWB*_ – ISI comparison factors; *p*_*2,1*_ – *p*-value for differences between two- and one-parameter linear fitting; *p*_*3,2*_ – *p*-value for differences between three- and two-parameter linear fitting; *PFS* – post firing silence; *SF* – silence factor; *S*_*RASP*_ – slope of linear fitting of rapid transient; *SWA* – slow wave amplitude; *SWA*_*min*_ – minimum slow wave amplitude. In PSTUT and PSWB, ${IS}{{I}}_{{i}}^{{\max }}$ indicates the maximum inter-spike interval and *ISI*_*i+1*_ indicates the immediately following inter-spike interval.

A persistent firing response with relatively equal inter-spike intervals denotes non-adapting spiking (NASP); in contrast, transients with a progressive increase or decrease of ISIs can be classified as adapting or accelerating spiking, respectively. To discriminate among several possible combinations of these firing patterns objectively and reproducibly, we devised a minimum information description criterion by comparing piecewise (segmented) linear regression models of increasing complexity. Specifically, non-adapting spiking (NASP) can be described by a single parameter, namely the (average) firing rate (*Y* = *c*). Similarly, fitting normalized inter-spike intervals versus normalized time with a (2-parameter) linear function *Y* = *aX* + *b* (with *a* > *0*) corresponds to adapting spiking (ASP.). Fitting data with a piecewise linear function

$${Y}=\{\begin{array}{cc}{{a}}_{{1}}{X}+{{b}}_{{1}} & {if}\,{X} < \frac{{{b}}_{2}-{{b}}_{1}}{{{a}}_{1}-{{a}}_{2}}\\ {{a}}_{2}{X}+{{b}}_{2} & {if}\,{X}\ge \frac{{{b}}_{2}-{{b}}_{1}}{{{a}}_{1}-{{a}}_{2}}\end{array}$$

corresponds to adapting-non-adapting spiking (ASP.NASP) when *a*_*1*_ > *0* and *a*_2_ = *0* (3 parameters), and to adapting-adapting spiking with different adaptation rates (ASP.ASP.) when both *a*_1_ > *0* and *a*_2_ > *0* (4 parameters). We only selected a model with more parameters if the fit relative to a less complex model improved in a statistically significant way. The significance threshold for the differences between one-parameter fitting (NASP) and two-parameter linear-regression fitting (ASP.) was conventionally set at 0.05. Furthermore, in order to avoid identifying very weak adaptations as ASP., a minimum threshold of 0.003 was used for the slope *a*_1_.

For each subsequent stage of comparison, we used Bonferroni-corrected *p*-values. Specifically, in order for a pattern with an adapting spiking transient (i.e. ASP.) to be qualified as ASP.NASP, the *p*-value must be less than 0.025. Similarly, the *p*-value for the differences between three-parameter piecewise-linear-regression fitting (ASP.NASP) and four-parameter piecewise-linear-regression fitting (ASP.ASP.) must be less than 0.016. Supplementary Fig. S1 shows examples of fitting spiking activity with linear regression and piecewise linear regression models. If adaptation was only observed in the first two or three ISIs in a longer train of spikes, and if the linear fitting of slope *a*_1_ exceeded 0.2, then this transient was classified as rapidly adapting spiking (RASP.) (see Fig. 1; cf. Pawelzik *et al*.^18^). For accelerating spiking (ACSP.), the linear fitting slope must be negative.

We defined transient stuttering (TSTUT.) as a short high-frequency (>25 Hz) cluster of action potentials (APs) followed by other distinctive activity. In addition, the first ISI after a TSTUT cluster must be 2.5 times longer than the last ISI of the cluster and 1.5 times longer than the next ISI (see Fig. 1; cf. Hamam *et al*.^27^). Under transient slow-wave bursting activity (TSWB.), a cluster of two or more spikes rides on a slow depolarization wave (>5 mV) followed by a strong slow after-hyperpolarization (AHP) (see Fig. 1; cf. Chevaleyre & Siegelbaum^28^). Persistent stuttering (PSTUT) was classified as firing activity with high-frequency clusters of APs separated by long silence intervals, moreover, the sum of its ratios to the preceding ISI and the following ISI is more than 5 (see Fig. 1; cf. Fuentealba *et al*.^29^; Price *et al*.^30^). Similarly, under persistent slow-wave bursting (PSWB) activity, these clusters of two or more tightly grouped spikes ride on slow depolarizing waves (>5 mV) followed by strong, slow AHPs^31,32^. The amplitude of the slow wave was determined as the difference between the threshold of initiation of the slow wave and the threshold of generation of the action potential at the top of the slow wave. Threshold level was defined as a point of fast rising of the membrane voltage (for slow wave initiation and action potential generation *dV/dt* should exceed 0.15 V s^−1^ and 20 V s^−1^, respectively). As exemplified above, the choices of firing-pattern identification parameters were consistent with literature reports of experimental results with similar activities.

Based on the aforementioned methods, we implemented a firing-pattern classification algorithm using the values of ISIs, delay, post-firing silence, and slow-wave amplitude as input data (for algorithmic details, see Supplementary Fig. S2).

### Statistical analysis

We explored pairwise correlations between 9 firing pattern elements and another 110 properties expressed in Hippocampome.org neuron types, including: primary neurotransmitter, the projecting (between sub-regions) or local (within sub-regions) nature of axonal and dendritic patterns; clear positive or negative expression of 98 molecular markers; high (top third) or low (bottom third) values for 10 electrophysiological properties^25^. To evaluate the correlations between these categorical properties, we used 2 × 2 contingency matrices with Barnard’s exact test^33^, which provides the greatest statistical power when row and column totals are free to vary^34^. We calculated *p*-values using the selected 2 × 2 tables, which had a total of more than 9 elements and in which the corresponding FPE was observed more than three times. As a result, a total of 155 correlations were analyzed. The results satisfying *p*-value cutoff of <0.05 and a false discovery rate (FDR) < 0.25^35^ were considered as statistically significant. The correlation analysis was implemented in MATLAB (MathWorks, Inc.).

We analyzed numerical electrophysiological data, such as the relationship between the width of an action potential and the minimum ISI using linear regression and histograms. Spike duration was measured as the width at half-maximal amplitude, as is most commonly defined^36^. Minimum inter-spike intervals (*ISI*_*min*_) were measured from the figures or extracted directly from tables or textual excerpts of the corresponding papers.

For cluster analysis of weighted categorical firing pattern data, we assigned weights to firing pattern elements according to the formula *W*_*e*_ = *(N* *−* *n*_*e*_*)/N*, where *W*_*e*_ is the weight of the element *e*, *n*_*e*_ is the number of cell types expressing firing pattern(s) with element *e*, *N* is the total number of cell types/subtypes, and *e* = {ASP., D., RASP., NASP, PSTUT, PSWB, SLN, TSUT., TSWB.}. We employed a two-step cluster analysis using the IBM SPSS Statistics 24 software. Silhouette measures of cohesion and separation greater than 0.5 indicated that the elements were well matched to their own clusters and poorly matched to neighboring clusters, and that the clustering configuration was appropriate.

Statistical data were expressed as mean ± standard deviation.

### Web portal and database representation of firing patterns and experimental conditions

Hippocampome.org provides access to morphological, molecular, electrophysiological, and connectivity information for 122 neuron types. The firing pattern data newly added and made freely available for download with this work include recording illustrations, the duration and amplitude of stimulation, digitized ISIs and firing pattern parameters (as comma-separated-value files), the complete solution compositions of the ACSF and of the micropipettes or patch pipettes, and the result of the firing pattern classification algorithm detailed above. Additional metadata is collected and displayed for all electrophysiological evidence in Hippocampome.org including the animal species (rat vs. mouse) and other details regarding the subject (inbred strain, age, sex, and weight, if reported), slice thickness and orientation, recording methods (intracellular microelectrode or variations of patch clamp), and temperature. Details of the implementation of the portal are presented in the Supplemental Information.

## Results

### From firing patterns to firing pattern phenotypes

Version 1.3 of Hippocampome.org contains suitable electrophysiological recordings for 90 of the 122 morphologically identified neuron types. Applying the firing pattern identification algorithm to these digitized data resulted in the detection of 23 different firing patterns. A given neuron type may demonstrate distinct firing patterns in response to different stimuli or conditions. The set of firing patterns exhibited by a given neuron type forms its firing pattern phenotype.

The simplest case consists of those neuron types that systematically demonstrate the same firing pattern independent of experimental conditions or stimulation intensity. These neuron types may still display quantitatively different responses to stimuli of various amplitudes (typically increasing their firing frequency upon increasing stimulation), but their qualitative firing patterns remain the same. We identified 37 such “individual/simple-behavior types” in Hippocampome.org, as exemplified by DG Basket cells with their NASP phenotype^37^.

In contrast to the above scenario, certain neuron types exhibit qualitatively distinct firing patterns in response to different amplitudes of stimulation. We identified 20 such “multi-behavior” types; for instance, medial EC Layer V-VI Pyramidal-Polymorphic cells demonstrate delayed non-adapting and adapting spiking^14^, or CA1 Neurogliaform projecting cells^30^ display adapting spiking and persistent stuttering at different stimulus intensities. The firing phenotypes of these neurons thus consist of the combinations of two firing patterns.

In a different set of cases, subsets of neurons from the same morphologically identified type display distinct firing patterns under the same experimental conditions (typically from the same study) in response to identical stimulation. These neuron types can thus be divided into electrophysiological subtypes. For example, of the CA3 Spiny Lucidum interneurons, some are adapting spikers whereas others are persistent stutterers^38^. In certain neuron types, one or more of the subtypes could also display multiple behaviors at different stimulation intensities. For instance, a subset of entorhinal Layer III Pyramidal neurons consists of non-adapting spikers and another subset switches from ASP.NASP at rheobase to RASP.ASP. at higher stimuli^14^. Of the 90 neuron types with firing patterns in Hippocampome.org, 22 could be divided into 52 electrophysiological subtypes. Notably, these included the principal neurons of most sub-regions of the hippocampal formation: CA3, CA1, and subiculum Pyramidal cells, entorhinal Spiny Stellate cells, but also several GABAergic interneurons such as dentate Total Molecular Layer (TML) cells^39^. Specifically, 8 neuron types yielded 18 subtypes exclusively demonstrating single behaviors; for 11 neuron types, at least one of the subtypes exhibited multi-behaviors, resulting in 13 multi-behavior subtypes and 13 additional single-behavior subtypes.

This meta-analysis is complicated by the variety of experimental conditions used in the published literature from which the electrophysiological data were extracted. Several differences in materials and methods could affect firing patterns above and beyond common species (rats vs. mice) or recording (patch clamp vs. microelectrode). For example, 30% of experimental traces were recorded from transverse slices, 24% from horizontal, 8% coronal, 29% mixed (e.g. “horizontal or semicoronal”), and 9% other directions (e.g. custom angles). Furthermore, pipettes were filled with potassium gluconate in 69% of cases, with potassium methylsulfate in 22%, and with potassium acetate in 9% (see e.g. Supplementary Table S2). While these different experimental conditions can affect membrane biophysics substantially^40^ and often quantitatively influence neuronal firing (e.g. changing the spiking frequency), occasionally they can also cause a qualitative switch between distinct firing patterns. A striking case is that of rat DG Granule cells, which have demonstrated transient slow-wave burst followed by silence in whole-cell recordings of horizontal slices from Sprague-Dawley animals^41^; delayed non-adapting spiking in whole-cell recording of transverse slices from Wistar animals^16^; or adapting spiking in intracellular recording of horizontal slices from Wistar animals^42^. Because the different firing patterns could be caused by the differences in experimental methods, we annotate a possible “condition-dependence,” but cannot conclusively categorize these cells as multi-behavior or subtypes. Most of the condition-dependent behaviors could be attributed at least in part to the occasional use of microelectrode instead of patch-clamp (now considered the preferred recording method) or the animal species as in the case of CA1 Horizontal Basket cells, which display adapting and non-adapting firing in rats and mice, respectively^19,43^.

Condition dependence can alter the firing patterns not only in cell types with single behaviors, such as MOPP cells^42,44^, but also in multi-behavior neuron types, such as CA1 Axo-axonic cells^18,45^. These cases account for 6 and 5 Hippocampome.org neuron types, respectively. Lastly, condition dependence may also be found in specific electrophysiological subtypes, whether they display single behaviors, such as CA1 Pyramidal neurons^28,43,46,47^ or multi-behavior, such as entorhinal Layer V Deep Pyramidal neurons^14,27,48^. These cases respectively account for 2 and 1 Hippocampome.org neuron types, in turn giving rise to 6 condition-dependent subtypes with single behaviors and 2 condition-dependent subtypes with multi-behavior. In general, types/subtypes with firing pattern recorded under diverse experimental conditions constitute only 16 percent of the total number of types/subtypes with available recordings.

**Figure 2:**
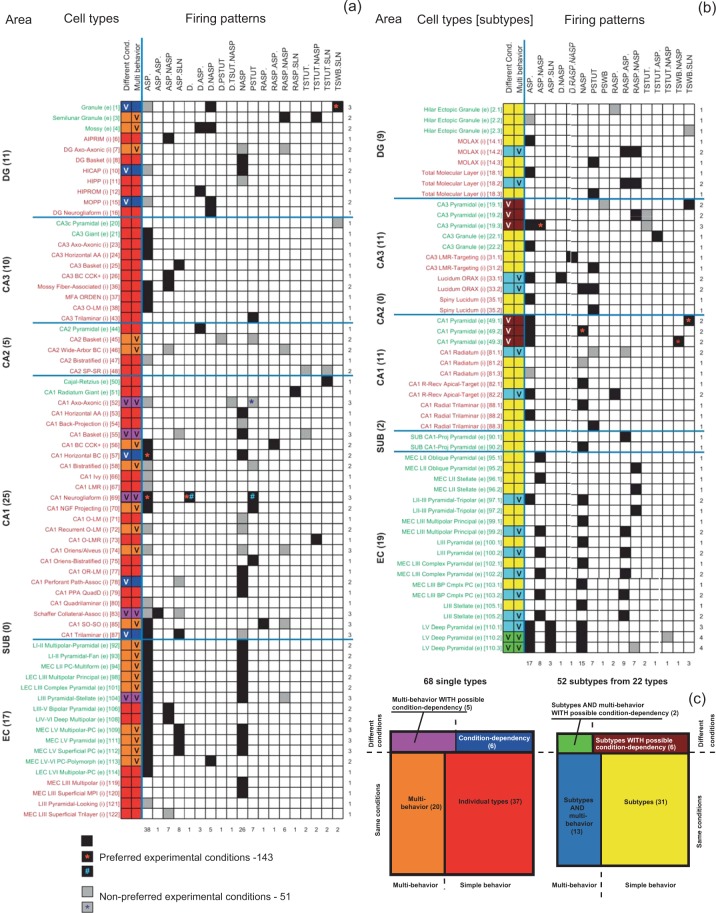
Identified firing patterns and firing pattern phenotypes complexity of neuron types (**a**) and subtypes (**b**). Online matrix: hippocampome.org/firing_patterns.php. Green and red cell type/subtype names denote excitatory (*e*) and inhibitory (*i*) neurons, respectively. FPP is firing pattern phenotype. The numbers in the brackets correspond to the order in which the cell types were presented in the Hippocampome.org (ver. 1.3). The orange asterisk denotes different experimental conditions. (**c**) Complexity of firing pattern phenotypes; percentages and ratios indicate occurrences of phenotypes of different complexity among 120 cell types/subtypes.

Figure 2 presents the full firing-pattern phenotypes of all 90 Hippocampome.org neurons, with available data in form of separate matrices for the 68 individual neuron types (Fig. 2a) and the 52 subtypes divided from the remaining 22 types (Fig. 2b). In both cases the simple behaviors constitute larger proportions than multi-behavior, with condition dependence only reported for a minority of types and subtypes (Fig. 2c). Across these neuron types/subtypes, 44 distinct phenotypes can be identified as unique combinations of firing patterns, excluding those that differ from others only by the absence of a detectable stable state in one of the firing patterns (like ASP. versus ASP.NASP or ASP.SLN). An interactive online version of these matrices is available at hippocampome.org/php/firing_patterns.php.

### Dissecting firing patterns into firing pattern elements across neuron types

**Figure 3:**
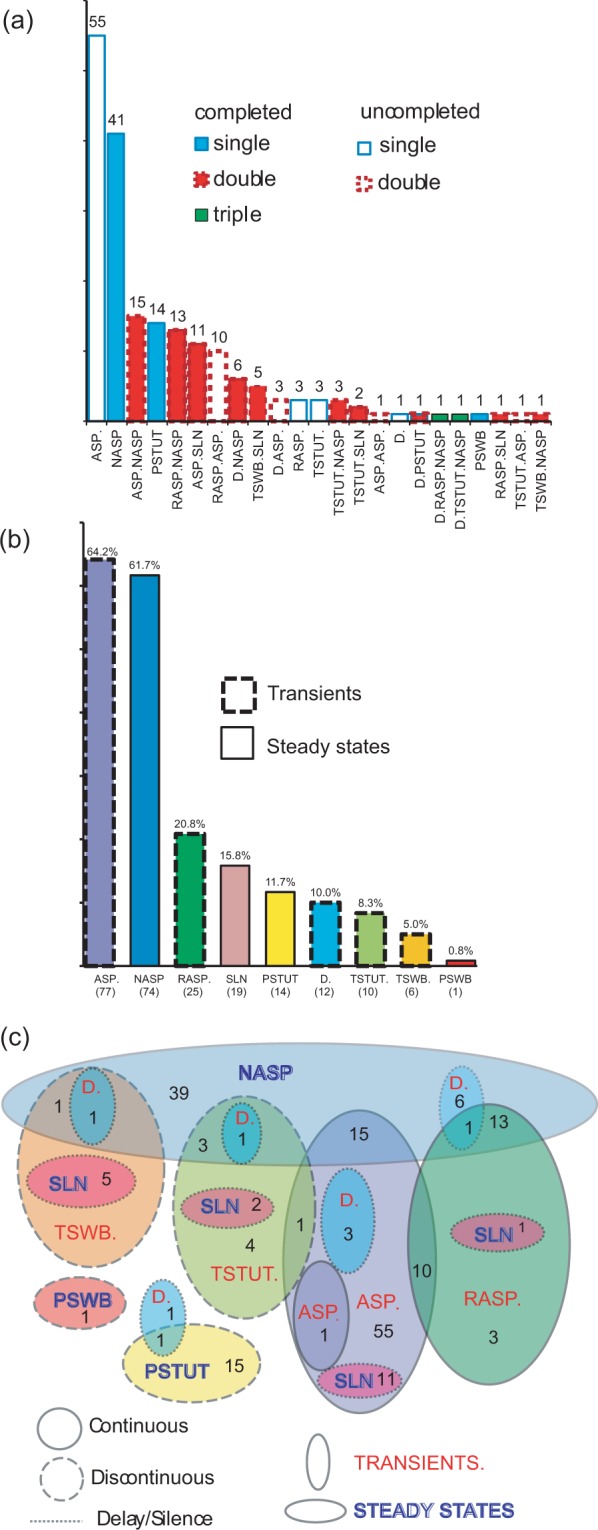
Occurrence of firing patterns, firing pattern elements and firing pattern phenotypes among the hippocampal formation neuron types. **(a)** Distribution of 23 firing patterns; total numbers are shown above the bars. **(b)** Distribution of 9 firing pattern elements; total numbers are in parentheses below and percentages of occurrence among 90 cell types are above the bars. **(c)** Relationships between firing pattern elements in the firing patterns of hippocampal neuron types. Numbers of cell types with distinctive firing patterns are indicated.

Firing patterns and firing pattern elements are also diverse with respect to their relative frequency of occurrence among hippocampal neuron types. Firing patterns can be grouped based on the number of elements comprising them, namely single (e.g., NASP or PSTUT), double (e.g. ASP.NASP or TSWB.SLN), and triple (D.RASP.NASP and D.TSWB.NASP) or based on whether they are completed (ASP.NASP, TSWB.SLN) or uncompleted, as in ASP., RASP.ASP., and TSTUT.ASP. (Fig. 3a). Of the nine firing pattern elements, the most frequent are ASP and NASP, while the least common are TSTUT, TSWB, and PSWB (Fig. 3b). Notably, accelerated spiking (ACSP) has not been reported in the rodent hippocampus although it is commonly observed in other neural systems, such as turtle ventral horn interneurons^49^ and motoneurons^50^.

The relationships between sets of firing pattern elements observed in hippocampal neuron types can be summarized in a Venn diagram with firing pattern elements represented as ellipses and the intersections thereof corresponding to complex firing patterns (Fig. 3c). This analysis highlights the following features: the four main firing transients (ASP., RASP., TSTUT., TSWB.) often end either with NASP or with SLN; ASP. is often preceded by RASP. and occasionally by TSTUT.; interrupted steady-state firings (PSTUT and PSWB) stand out as a separate group; and delay (D.) most often precedes NASP. Fifteen of possible 38 completed firing patterns were discovered in the literature for morphologically identified hippocampal neuron types (Table S3 in Supplementary Information).

### Classification and distribution of firing pattern phenotypes

**Figure 4:**
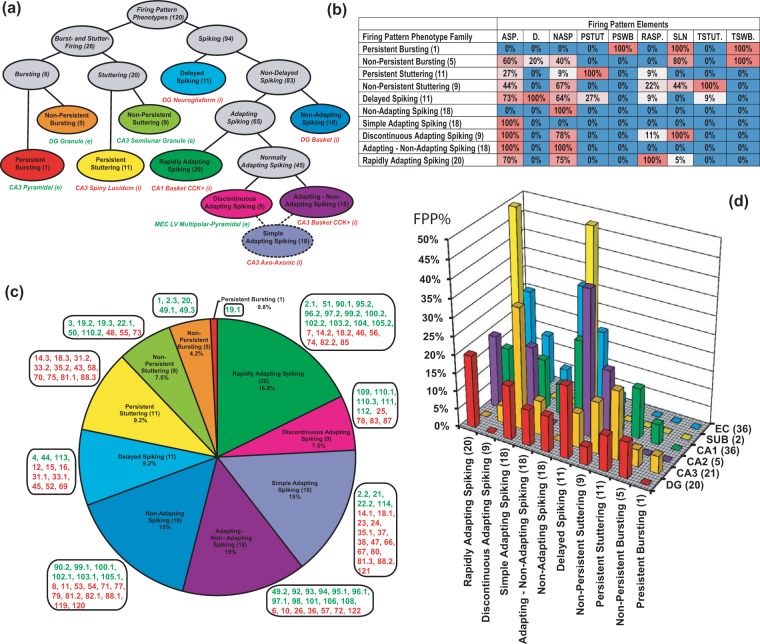
Firing-pattern phenotype families from 120 neuron types/subtypes. **(a)** Hierarchical tree resulting from two-step clustering of weighted firing pattern elements with representative examples of cell types/subtypes that belong to one of the corresponding firing-pattern phenotype families. Note that the simple adapting spiking pattern (ASP. only) constitutes a “spurious” phenotype of uncompleted adapting spiking (15%), for which the steady state (SLN or NASP) was not determined. **(b)** Percentage of occurrence of firing-pattern elements in families of firing pattern phenotypes. **(c)** Relative proportions of firing-pattern phenotype families among neuron types/subtypes. Green and red numbers represent excitatory and inhibitory cell types/subtypes as enumerated in Fig. 2. **(d)** Distribution of firing-pattern phenotype families in sub-regions of the hippocampal formation. FPP% is percentage of expression of families of firing pattern phenotypes.

In order to classify the 44 unique firing pattern phenotypes observed in the hippocampal formation, we weighted the constituent firing pattern elements according to the frequency of occurrence among 120 neuron types and electrophysiological subtypes (see *Methods*). As a result, infrequent firing pattern elements (PSWB, TSTUT and TSWB) received high weights (0.99, 0.95 and 0.93, respectively), very frequent elements (ASP and NASP) were assigned low weights (0.42 and 0.41), and common elements (D, RASP, PSTUT and SLN) obtained intermediate weights (0.90, 0.80, 0.88 and 0.87). Two-step cluster analysis identified ten firing pattern families as leaves of a seven-level hierarchical binary tree (Fig. 4a). At the highest level, hippocampal neuron types and subtypes are divided into two major groups: those with spiking phenotypes (78%) and those with interrupted firing phenotypes (22%). The latter are separated into bursting (6%) and stuttering (16%), and each of these is subdivided into persistent and non-persistent families. A first group of the neuron types with spiking phenotypes is distinguished based on delay (9% of cell types). The remaining neuron types split into adapting (54%) and non-adapting phenotypes (15%). The adapting group consists of neuron types with rapidly adapting phenotypes (18%) and normally adapting (36%) phenotypes. Among the normally adapting group, the following phenotypes can be distinguished: discontinuous adapting spiking (6%) with ASP.SLN pattern, adapting-non-adapting spiking (15%) with ASP.NASP patterns, and a last “spurious” phenotype of uncompleted adapting spiking (15%) with ASP. pattern only, for which the steady state (SLN or NASP) was not determined. This division of the adapting spiking groups reflects differences in adaptation rates, duration, and subsequent steady states.

This analysis also highlights the most distinguishing firing pattern elements of each family (Fig. 4b). In particular, D. is the defining element for delayed spiking, PSTUT for persistent stuttering, ASP. and SLN for discontinuous adapting spiking. Each of the four major elements of interrupted firing patterns (PSWB, PSTUT, TSWB. and TSTUT.) is observed in a single firing pattern phenotype (persistent bursting, non-persistent bursting, persistent stuttering, and non-persistent stuttering, respectively). Other firing pattern elements (D., RASP., ASP., NASP, and SLN) appear in several firing pattern phenotypes. The proportions of non-defining firing pattern elements range from 5% to 83%.

The families of firing pattern phenotypes are differentially distributed within the set of 120 neuron types/subtypes (Fig. 4c). Certain phenotype families are associated with excitatory neuron types, either exclusively (e.g. persistent bursting and non-persistent bursting) or predominantly (non-persistent stuttering, rapidly adapting, and adapting-non-adapting spiking). Conversely, persistent stuttering, delayed spiking, non-adapting spiking and simple adapting spiking are phenotypes composed largely by inhibitory neuron types. The discontinuous adapting spiking phenotype has relatively balanced proportions of excitatory and inhibitory neuron types.

The firing pattern phenotypes also have different distributions among the sub-regions of the hippocampal formation (Fig. 4d). Among CA1 neuron types, the persistent stuttering (16%), non-adapting (24%), simple adapting (16%), and rapidly adapting spiking (13%) phenotypes are more common than other phenotypes; in DG, the most expressed phenotypes are delayed (20%), rapidly adapting (20%), and simple adapting spiking (15%); in EC, ASP-NASP (61%), discontinuous ASP. (11%), RASP. (28%), and NASP (19%) occur more often than other phenotypes.

### Usage of information from Hippocampome.org

#### Searching and browsing

**Figure 5:**
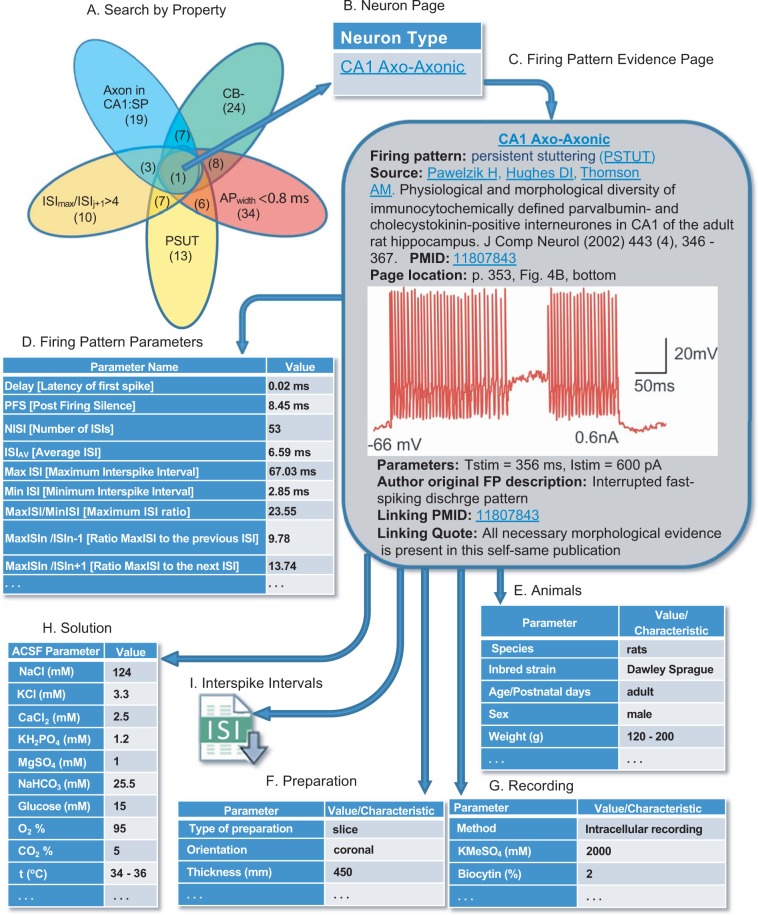
Hippocampome.org enables searching neuron types by neurotransmitter; axon, dendrite, and soma locations; molecular expression; electrophysiological parameters; input/output connectivity; firing patterns, and firing pattern parameters. **(a)** Sample query for calbindin-negative neuron types with axons in CA1 stratum pyramidale, *AP*_*width*_ < 0.8 ms, PSTUT firing, and ratio of maximum ISI to the next ISI greater than 4. Numbers in parentheses indicate the number of neuron types with the selected property or specific combination of properties. **(b)** Search results are linked to the neuron page(s). **(c)** The neuron page is linked to the firing pattern evidence page. Original data extracted from Pawelzik *et al*.^18^. All firing patterns parameters **(d)**, experimental details including information about animals **(e)**, preparations **(f)**, recording method and intra-pipette solution **(g)**, as well as ACSF composition **(h)** can be displayed. **(i)** Downloadable comma-separated-value file of inter-spike intervals.

The addition of firing pattern data to Hippocampome.org extends opportunities for broad-scope analytics and quick-use checks of neuron types. Similar to morphological, molecular, and biophysical information, firing patterns and their parameters can be browsed online with the interactive versions of the matrices presented in Fig. 2 (hippocampome.org/php/firing_patterns.php), along with an accompanying matrix to browse the stimulation parameters (duration and intensity) and the firing pattern parameters (delay, number of inter-spike intervals, etc.). Moreover, all classification and analysis results reported here can be searched with queries containing AND & OR Boolean logic using an intuitive graphical user interface (see Hippocampome.org → Search → Neuron Type). The integration within the existing comprehensive knowledge base enables any combination of both qualitative (e.g. PSTUT) and quantitative (e.g. ${IS}{{I}}_{{i}}^{{\max }} > 4\,{IS}{{I}}_{{i}+1}$) firing pattern properties, with molecular (e.g. calbindin-negative), morphological (e.g. axons in CA1 pyramidal layer), and biophysical (e.g. action potential width < 0.8 ms) filters (Fig. 5). For example, of 13 neuron types with persistent stuttering, in 7 the largest inter-spike interval (*ISI*^*max*^_*i*_) is more than 4 times longer than the subsequent ISI (*ISI*_*i+1*_). When adding the other three selected criteria, the compound search leads to a single hit: CA1 Axo-axonic neurons (Fig. 5a). Clicking on this result leads to the interactive neuron page (Fig. 5b) where all information associated with a given neuron type is logically organized, including synonyms, morphology, biophysical parameters, molecular markers, synaptic connectivity, and firing patterns. Every property on the neuron pages and browse matrices, including firing patterns and their parameters, links to a specific evidence page that lists all supporting bibliographic citations, complete with extracted quotes and figures (Fig. 5c). The evidence page also contains a table with all corresponding firing pattern parameters (Fig. 5d), experimental details including information about animals (Fig. 5e), preparations (Fig. 5f), recording method and intra-pipette solution (Fig. 5g), ACSF (Fig. 5h), and a downloadable file of inter-spike intervals (Fig. 5i).

The portal also reports, when available, the original firing pattern name descriptions used by the authors of the referenced publication (Hippocampome.org → Search → Original Firing Pattern) and provides links to corresponding published models from ModelDB (https://senselab.med.yale.edu/modeldb/).

#### Statistical analysis of categorical data

**Figure 6:**
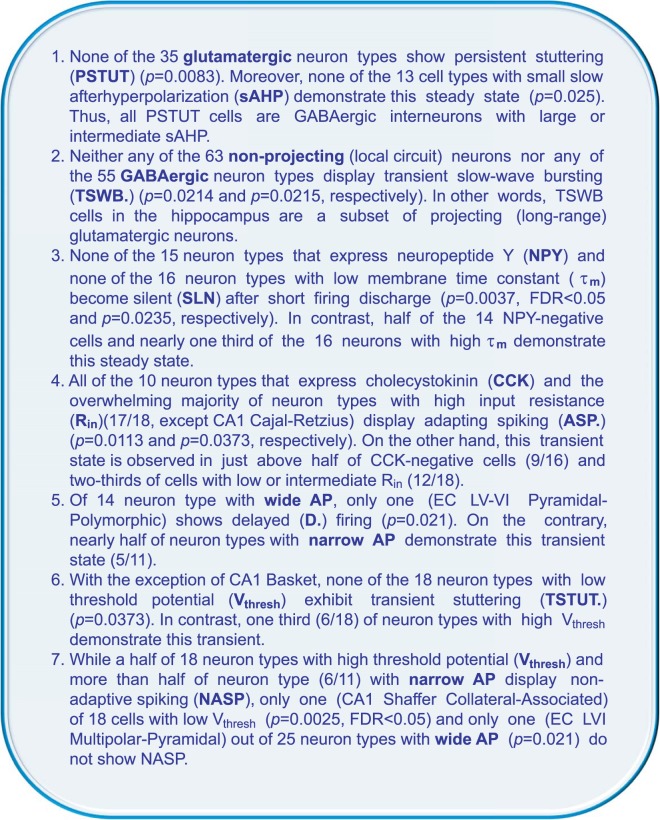
Examples of statistically significant correlations between firing pattern elements and known molecular, morphological and electrophysiological properties in hippocampal neurons. The *p* values are computed using Bernard’s exact test for 2 × 2 contingency tables and satisfy FDR < 0.25 (see *Methods*).

Firing pattern information more than doubles the Hippocampome.org knowledge base capacity to over 27,000 pieces of knowledge, that is, associations between neuron types and their properties. This extension allows for the confirmation of known tendencies and unearthing hidden relationships between firing patterns and molecular, biophysical, and morphological data in hippocampal neurons, which are otherwise difficult to find amongst the large body of literature. We computed *p*-values using Bernard’s exact test for 2 × 2 contingency tables. Comparisons of observable firing pattern elements, with molecular markers expression, electrophysiological parameters, primary neurotransmitter, and axonal projecting properties, with *p*-values less than 0.05 and false discovery rates less than 0.25, ended with 29 statistically significant correlations. Several interesting examples of such findings are presented in Fig. 6. For instance, adapting spiking (ASP.) tends to co-occur with expression of cholecystokinin (*p* = 0.0113 with Barnard’s exact test from all n = 26 pieces of evidence; see Lee *et al*.^51^ as an example); moreover silence (SLN) after short firing discharge is not observed in neuron types with low (lower tercile of) membrane time constant (n = 32, *p* = 0.0235).

#### Analysis of numerical electrophysiological data

**Figure 7:**
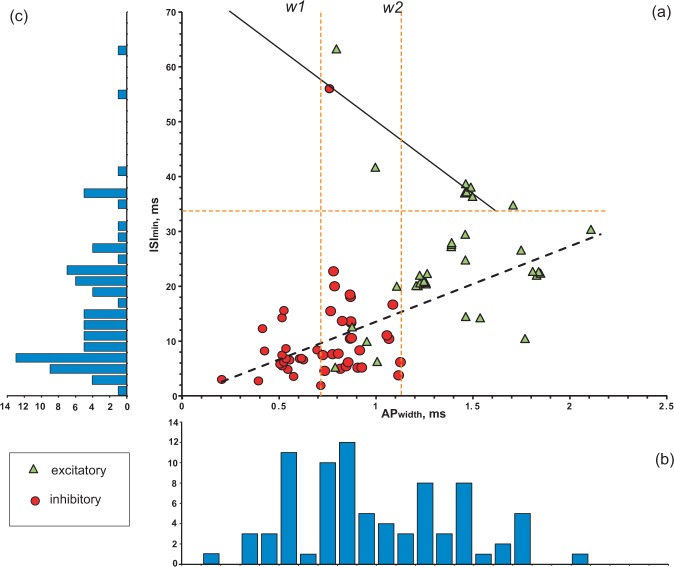
Relationships between the width of action potentials (*AP*_*width*_) and minimum of inter-spike intervals (*ISI*_*min*_) for 84 neuron types and subtypes. **(a)** *AP*_*width*_ *− ISI*_*min*_ scatter diagram with results of linear regression. Green triangles and red circles indicate excitatory and inhibitory neurons, respectively. Dashed orange lines: horizontal line separates neurons with slow spikes from neurons with fast and moderate spikes; vertical lines (*w*1 and *w2*) separate neurons with narrow, medium and wide action potentials. Black lines: solid line shows linear fitting for slow spike neurons with a function *Y* = *−26.72X* + *76.42* (*R*^*2*^ = 0.83); dashed line shows general linear fitting for fast and moderate spike neurons with a function *Y* = 1*3.79X* *−* *0.05* (*R*^*2*^ = 0.52). **(b)** *AP*_*width*_ histogram. **(c)** ISI histogram.

The extracted quantitative data allow one to study the relationship between firing pattern parameters and membrane biophysics or spike characteristics, such as the correlations between minimum inter-spike intervals (*ISI*_*min*_) and action potential width (*AP*_*width*_). We analyzed these two variables in the 81 neuron types and subtypes for which both measurements are available (Fig. 7). The scatter plot of *AP*_*width*_ against *ISI*_*min*_ reveals several distinct groupings (Fig. 7a), and the corresponding histograms (Fig. 7b,c) demonstrate poly-modal distributions. The horizontal dashed line (*ISI*_*min*_ = 34 ms) separates 9 neurons with slow spikes (all excitatory except one) from 72 neurons (61% of which are inhibitory) with fast and moderate spikes. The latter group shows a general trend of *ISI*_*min*_ rise with increasing *AP*_*width*_ (black dashed line in panel A). This trend was adequately fit with a linear function *Y* = *13.79X* *−* *0.05* (*R* = 0.72; *p* = 0.03). Neuron types with slow spikes demonstrate the opposite trend, which was fit with a decreasing linear function *Y* = *−*2*6.7*2*X* + *76.42* (*R* = −0.91, *p* = 10^−6^). Furthermore, the neuron types can be separated by spike width. The vertical dashed lines *w*1 (*AP*_*width*_ = 0.73 ms) and *w*2 (*AP*_*width*_ = 1.12 ms) separate neuron types with narrow, medium and wide action potentials. The group of neuron types with narrow spikes (n = 22) includes only inhibitory neurons, which have *AP*_*width*_ in the range from 0.20 to 0.73 ms (0.54 ± 0.12 ms). In contrast, the group of neuron types with wide spikes (n = 28) contains only excitatory neurons with *AP*_*width*_ in the range from 1.13 to 2.10 ms (1.49 ± 0.23 ms). The group of neuron types with medium spikes (n = 31), with *AP*_*width*_ range from 0.74 to 1.12 ms (0.89 ± 0.12 ms), includes a mix of inhibitory (74%) and excitatory (26%) neurons.

Among the 22 neuron types/subtypes from the group with *AP*_*width*_ < 0.72 ms, 13 demonstrated so-called fast spiking behavior, which is distinguished by narrow spikes, high firing rate, and the absence or weak expression of spike frequency adaptation^52^. Besides these common characteristics, however, their firing patterns vary broadly even from a qualitative standpoint. Five of these 13 neuron types belong to the PSTUT family, namely CA3 Trilaminar^53^, CA3 Aspiny Lucidum ORAX^54^, CA2 Basket^17^, CA1 Axo-axonic^18^, and CA1 Radial Trilaminar^19^. Three types belong to the NASP family: DG Basket^37^, CA1 Horizontal Axo-axonic^19^, and MEC LIII Superficial Multipolar Interneuron^55^. Two types, CA3 Axo-axonic^56^ and CA2 Bistratified^17^, belong to the simple adapting spiking family; two types, DG HICAP^39^ and DG AIPRIM^16^, belong to the ASP-NASP family; and lastly CA1 Basket^51^ belongs to non-persistent stuttering family.

Additionally, firing pattern families are unequally distributed among the groupings revealed by the above analysis. Persistent and non-persistent stuttering families and non-persistent bursting phenotypes are composed entirely of neuron types with narrow and medium fast/moderate spikes. Conversely, the rapidly adapting – non-adapting spiking phenotype is represented solely by neurons with spikes of intermediate width.

## Discussion

Neurons differ from each other by morphological and molecular features including the diversity and distribution of ion membrane channels in somata and dendrites. These intrinsic properties determine important physiological functions such as excitability, efficacy of synaptic inputs^57–59^, shapes of individual action potentials and their frequency^36^, and temporal patterns^60,61^.

In the neuroscience literature, the firing patterns of neuronal activity are commonly used to characterize or identify groups of neurons. Examples include descriptions of “strongly adapting, normally adapting, and nonadapting cells”^39^; “fast-spiking and non-fast-spiking” interneurons^62^; “late spiking” cells^63^; “stuttering interneurons”^64^; “bursting” and “non-bursting” neurons^65,66^; “regular spiking, bursting, and fast spiking”^67^, and many more. However, it has until now remained challenging to integrate these characterizations across different laboratories and studies besides largely qualitative summaries.

In this study, we show that a quantitative, data-driven methodology based on the analysis of transients and steady states of evoked spiking activity can meaningfully classify the firing patterns of hippocampal neuronal types. This work is a further development of the effort initiated by the Petilla Interneuron Nomenclature Group^23^, which was applied to firing patterns in cortical neurons^22,24^. At the same time, this work demonstrates the feasibility of systematic, comprehensive meta-analysis of electrophysiological data from the published literature. This is especially important as a necessary approach to help link and interpret the growing information from centralized, large-scale, “industrial” neuroscience projects^68–70^ with the distributed accumulation of data in traditional research laboratories^71^.

From the electrophysiological recordings of 90 neuron types in the rodent hippocampus, we identified 23 firing patterns, 15 of which were completed, that is, included both transient(s) and putative steady state components (see Figs. 2, 3). Taking into consideration the firing pattern information enables a possible refinement of neuron type delineation by identifying 52 putative electrophysiological subtypes among 22 neuron types. Subsequent two-step cluster analysis allows for the clear distinguishing of 9 unique (plus one spurious) families of 44 firing pattern phenotypes among 120 neuron types and putative subtypes. Notwithstanding the focus of the present research on the hippocampal formation, the firing pattern classification framework introduced with this study can be readily applied to spiking activity of neurons from other brain regions.

The two firing pattern families characterized by bursting phenotypes (transient and persistent) are comprised of excitatory neurons, while the persistent stuttering family only included inhibitory neurons. However, the majority of phenotype families are mixed between putatively glutamatergic and GABAergic types (Fig. 4c). Thus, the identification of a firing pattern phenotype by itself is a useful but in most cases insufficient attribute for a reliable categorization of excitatory and inhibitory neurons.

The frequency of discharges is an important characteristic of neuronal communication. Many neuron types, especially interneurons, show fast spiking behavior: they are capable of firing at high frequencies (200 Hz or more) with little decrease in frequency during prolonged stimulation^36,52^. Spike frequency correlates with electrophysiological characteristics, such as action potential duration or fast AHP amplitude^24^. Fast spiking neurons typically have narrow action potentials and high-amplitude fast AHP^36^. Our correlation analysis of Hippocampome.org data reveals that transient stuttering (TSTUT.) is not typical for cells with extremely high-amplitude fast AHPs and delayed firing (D.) is not characteristic for neuron types with wide action potentials (Fig. 6). Interestingly, plotting *ISI*_*min*_ against *AP*_*width*_ for all neuron types with relatively faster firing (maximum frequencies higher than ~30 Hz) and for all neuron types with slower firing (maximum frequencies lower than 29 Hz) reveals opposite, statistically significant linear relationships (Fig. 7a).

Firing pattern phenotypes of central mammalian neurons are determined by biophysical properties associated with expression and distribution of several types of Ca^2+^ and K^+^ channels, which modulate specific ion currents^36,72,73^ and may correlate with expression of other molecular markers^21,23,74^. Despite the relative sparsity of molecular marker information, analysis of the correlations between firing patterns and other neuronal properties revealed novel interesting relationships in hippocampal neuron types (Fig. 6).

Firing patterns play important roles in neural networks including the representation of input features, transmission of information, and synchronization of activity across separate anatomical regions or distinct cell assemblies. Although single spikes can provide temporally precise neurotransmitter release, this release usually has low probability in central synapses. Neurons can compensate for the unreliability of their synapses by transmitting signals via multiple synaptic endings or repeatedly activating a single synapse^75^. Thus, a brief, high-frequency sequence of action potentials may cross a synapse more reliably, increasing the likelihood of a postsynaptic spike^76^. This can also be affected by short-term synaptic plasticity^77,78^, which varies with age and with the identity of pre- and post-synaptic neurons. Moreover, single burst of action potentials in CA3 axons (Schaffer collaterals) can induce robust and stable long-term potentiation at synapses on CA1 pyramidal neurons, provided that the postsynaptic depolarization triggers a dendritic spike^79^. Recent results have also revealed that single bursts in DG granule cells may selectively alter specific functional components of the downstream circuit, such as feedforward inhibitory interneurons^80^.

Experimental studies provide strong evidence that different brain circuits employ distinct schemes to encode and propagate information^81^: while information relay by isolated spikes is insignificant for the acquisition of recent contextual memories in the hippocampus, it is essential for memory function in the medial prefrontal cortex. However, even within the hippocampus, different neuronal circuits may employ distinct coding schemes by relying on isolated spikes or bursts of spikes for execution of critical functions^81^. Indeed, distinct sub-regions of the hippocampal formation show differential distributions of spiking, bursting, and stuttering firing pattern phenotypes (Fig. 4).

In this study, the phenotyping of most types of neurons was based on the quantitative analysis of data extracted from single (or limited numbers of) figures exemplified neuronal electrical activity in relevant publications. Until neuroscience switches to the systematic deposition of all firing traces recorded and analyzed for a given publication to public repositories, such representative illustrations, however limited, constitute a fairly accurate reflection of the communal knowledge about neuronal physiology in particular neural system. Thus, our approach is based on the statistical quantification of integrated data presented in the literature.

The findings presented in this report resulted from the analysis of firing patterns in response to depolarizing current. To this date, this is by far the most common experimental protocol for characterizing the neuronal input-output function. Nevertheless, different types of neurons also exhibit distinct responses to hyperpolarization, as well as to its termination. For example, several neuron types described in Hippocampome.org demonstrate rebound spiking: CA1 Trilaminar^19,82^, CA1 Back-Projection^83^, CA1 O-LM^82^, CA1 SO-SO^18^, MEC LIII Multipolar Interneuron^55^, MEC LII Stellate^14^, MEC LII Oblique Pyramidal^14^. Such neuronal behaviors, owing to the hyperpolarization-activated cation current (*h*-current), may play an important role in hippocampal rhythmogenesis^84^ and could be locally modulated by activity-dependent changes in intrinsic excitability^85^. It will therefore be interesting to extend the current firing pattern phenotyping by considering these additional neuronal properties in future work.

The information on firing patterns of neuron types further expands the rich knowledge base of neuronal properties Hippocampome.org, which already contained information on morphology, molecular marker expression, connectivity, and other electrophysiological characteristics^25^. Computation of the potential connectivity map of all known 122 neuron types by supplementing available synaptic data with spatial distributions of axons and dendrites enabled the reconstruction of a circuitry containing more than 3200 putative connections^86^.

Modern experimental techniques allow researchers to conduct detailed morphological analysis^87^ and digital reconstructions of neurons^88^, collect biophysical and electrophysiological data, and develop complex multi-compartmental models in order to study synaptic efficacy^59^, synaptic^89^ and dendritic integration^90^, dendritic input discrimination capabilities^91^ and other neuronal properties. The main feature of the Hippocampome.org knowledge base is evidence-linked information about location of dendrites and axons in different sections and layers of the hippocampus, which served as the basis for neuron type classification. Similarly, we identified, extracted, and organized basic electrophysiological parameters and firing pattern information, which gave us the opportunity for quantitative firing-pattern phenotyping and comprehensive coverage of intrinsic diversity of neuronal types with simple models^92^. We developed compact multi-compartment models with up to four compartments, enabling synaptic integration of spatially segregated input pathways while significantly reducing the computational cost of large-scale network simulations^93^. More detailed morphological, electrophysiological and molecular information for every neuron type can be found on the provided links to the cited articles, as well as to the models published in ModelDB^94^. Among them are multicompartment models that consider the details of the morphology and biophysical properties, including distributions of specific ion channels and synaptic inputs, such as models of CA1 Pyramidal cells with non-uniformly distributed A-type potassium and hyperpolarization-activated channels^69,95^ based on experimental observations.

This ongoing accumulation of data and knowledge makes Hippocampome.org a powerful tool for building real-scale models of the entire hippocampal formation, thus substantially expanding the potential scope of recent advances in this regard^95^. More generally, such knowledge bases are playing an increasingly important role in neuroscience research by fostering computational analyses and data-driven simulations.

## Supplementary information

Supplementary Information.

## Data Availability

The datasets generated and/or analyzed during the current study are available at the Hippocampome.org.
