# Supplementary material for: Quantitative firing pattern phenotyping of hippocampal neuron types
Source: Sci Rep. 2019 Nov 29;9:17915. doi: 10.1038/s41598-019-52611-w (PMC6884469; doi:10.1038/s41598-019-52611-w)
Supplement: Supplementary file 1 — Supplementary Information. [file 41598_2019_52611_MOESM1_ESM.docx]

**Quantitative firing pattern phenotyping of hippocampal neuron types**

Alexander O. Komendantov, Siva Venkadesh, Christopher L. Rees, Diek W. Wheeler, David J. Hamilton, Giorgio A. Ascoli

Krasnow Institute for Advanced Study, George Mason University, 4400 University Drive, MS 2A1, Fairfax, Virginia 2230

**Supplementary Information**

Supplementary Methods

- Examples of experimental solutions (Suppl. Table S1 and S2)
- Examples of fitting of spiking activity (Suppl. Fig. S1)
- Algorithm of firing pattern identification (Suppl. Fig. S2)
- Implementation of the Web portal

Supplementary Results

- Completed firing patterns in hippocampal and other neurons (Suppl. Table S3)

Supplementary References

**Supplementary Methods**

**Examples of experimental solutions**

**Supplementary Table S1. Representative examples of artificial cerebrospinal fluids**

| NaCl (mM) | KCl (mM) | CaCl_2_ (mM) | NaH_2_PO_4_ (mM) | KH_2_PO_4_ (mM) | MgCl_2_ (mM) | MgSO_4_ (mM) | NaHCO_3_ (mM) | Glucose (mM) | t (^⚬^ C) | Pct. | Sample Reference |
| --- | --- | --- | --- | --- | --- | --- | --- | --- | --- | --- | --- |
| 126 | 3 | 2 | 1.25 |  |  | 2 | 26 | 10 | 34 | 30.0% | Canto and Witter^13^ |
| 124 | 3.3 | 2.5 |  | 1.2 |  | 1 | 25.5 | 15 | 34-35 | 11.3% | Ali and Thomson^97^ |
| 120 | 3.3 | 1.8 | 1.23 |  |  | 1.2 | 25 | 10 | 32-34 | 6.9% | Mott *et al.*^39^ |
| 130 | 3.5 | 2.5 | 1.25 |  | 1.5 |  | 24 | 10 | 32-34 | 6.5% | Tricoire *et al.*^19^ |
| 126 | 2.5 | 2 | 1.25 |  | 2 |  | 26 | 10 | 34-37 | 6.5% | Zemankovics *et al.*^43^ |
| 126 | 3 | 2 | 1.25 |  |  | 2 | 24 | 10 | 34-35 | 5.3% | Buhl *et al.*^45^ |
| 125 | 2.5 | 2 | 1.25 |  | 1 |  | 25 | 25 | 35-37 | 4.5% | Lübke *et al.*^16^ |
| 124 | 5 | 2 | 1.25 |  |  | 2 | 26 | 10 | 33-35 | 2.8% | Hamam *et al.*^48^ |
| 124 | 3 | 2.5 | 1.23 |  |  | 1.2 | 26 | 10 | 30 | 2.0% | Williams et al., 2007^41^ |

**Abbreviations:** Pct. – percentage of firing pattern recordings for which this solution was used.

**Supplementary Table S2. Representative examples of solutions for patch pipette and micropipette filling**

| KMeSO_4_ (mM) | KAc (mM) | KGlu (mM) | KCl (mM) | NaCl (mM) | MgCl_2_ (mM) | EGTA (mM) | Mg-ATP (mM) | Na_2_-ATP (mM) | GTP/ Na_1-3_-GTP (mM) | PCr/ Na-PCr (mM) | Biocytin (%) | Lucifer yellow (%) | Pct. | Sample Reference |
| --- | --- | --- | --- | --- | --- | --- | --- | --- | --- | --- | --- | --- | --- | --- |
|  |  | 110 | 10 |  |  |  | 4 |  | 0.3 | 10 | 0.5 |  | 29.1% | Canto and Witter^13^ |
|  |  | 130 | 7 |  |  |  | 2 |  | 0.3 |  | 0.2-0.4 |  | 6.9% | Mott *et al.*^39^ |
|  |  | 150 |  |  | 3 | 0.5 | 2 |  | 0.3 |  | 0.2 |  | 6.5% | Tricoire *et al.*^19^ |
|  |  | 126 |  |  |  |  | 4 |  | 0.3 | 10 | 0.5 |  | 4.9% | Price *et al.*^30^ |
|  |  | 90 | 27.4 | 1.8 | 1.7 | 0.05 | 2 |  | 0.4 | 10 | 0.03 |  | 2.8% | Armstrong *et al.*^44^ |
|  |  | 120 | 20 |  | 2 | 0.1 |  | 2 | 0.3 |  | 0.5 |  | 2.0% | Hemond *et al.*^15^ |
| 140 |  |  |  | 4 |  | 0.2 | 4 |  | 0.3 | 10 |  |  | 2.0% | Williams *et al.*^41^ * |
| 2000 |  |  |  |  |  |  |  |  |  |  | 2 |  | 11.3% | Ali and Thomson^99^* |
| 1500 |  |  |  |  |  |  |  |  |  |  | 2 |  | 5.3% | Buhl *et al.*^45^* |
|  | 1000 |  |  |  |  |  |  |  |  |  | 3 |  | 3.2% | Sik *et al.*^83^* |
|  | 4000 |  | 10 |  |  |  |  |  |  |  |  | 4 | 2.8% | Lacaille *et al.*^100^* |

**Abbreviations:** KAc – potassium acetate (KCH_3_COO); KGlu – potassium gluconate; KMeSO_4_ – potassium methylsulphate (CH_3_KSO_4_); PCr – phosphocreatine; Pct. – percentage of firing pattern recordings for which this solution was used; 10 mM HEPES (4-(2-hydroxyethyl)-1-piperazineethanesulfonic acid) was used in all patch pipette solutions. Asterisks indicate examples of micropipette solutions.

**
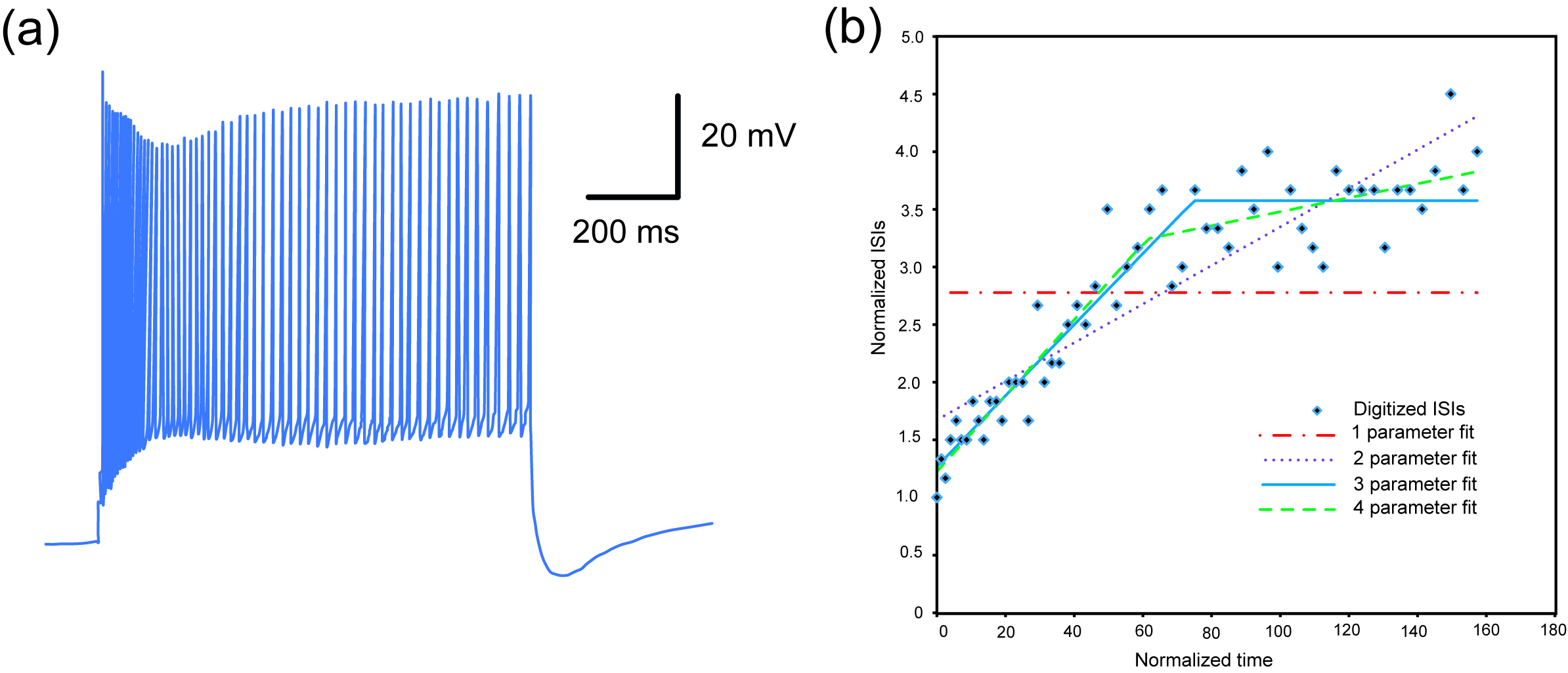
**

**Supplementary Figure S1.** **Examples of fitting of spiking activity with linear regression and piecewise linear regression models.** **(a)** Responses to current injection of a DG aspiny interneuron with axonal projection to the inner molecular layer (AIPRIM in Hippocampome.org) (Original data extracted from Lübke *et al.*^16^). **(b)** Fitting of digitized experimental data with different models.

1 parameter fit is a constant function *Y=2.78*;

2 parameter fit is a linear function *Y=0.017X+1.67*;

3 parameter fit is a piecewise linear function *$Y=\left\{\begin{matrix} 0.031X+1.27 if X<74.9 \\ 3.58 if X\geq74.9 \end{matrix}\right.$;*

4 parameter fit is a piecewise linear function *$Y=\left\{\begin{matrix} 0.033X+1.23 if X<61.3 \\ 0.006X+2.87 if X\geq61.3 \end{matrix}\right.$.*

Based on *p*-values, the firing pattern was identified as adapting-non-adapting spiking (ASP.NASP): *p*_2,1_ < 0.05 (*p*_2,1_ =1.26^.^10^-10^), *p*_3,2_< 0.025 (*p*_3,2_=2.7^.^10^-3^ ), *p*_4,3_> 0.016 (*p*_4,3_=5.5^.^10^-2^ ). *p*_2,1_, *p*_3,2_, *p*_4,3_ – p-values of differences between 2 parameter fit and 1 parameter fit, 3 parameter fit and 2 parameter fit, 4 parameter fit and 3 parameter fit, respectively.

**Algorithm of firing pattern identification**

Firing pattern elements were identified based on calculated characteristics of responses (Table 1 in *Methods*). First, it was determined whether the pattern contained a delay (D.), then whether it contained a TSTUT. or TSWB. The remaining ISIs were processed using the described statistical test to identify spike frequency adaptation (ASP., ASP.NASP, ASP.ASP.) by fitting the sequence of intervals with a piecewise linear function. In the case of an incomplete pattern or an insufficient number of ISIs to perform the test, the presence of post firing silence (SLN) was checked. If the test did not identify the pattern containing the adaptation, then the firing pattern was checked for the presence of PSTUT or PSWB, and then for NASP or RASP. If rapid adaptation was detected, the cycle with the statistical test was performed again on the remaining ISIs. The algorithm terminated upon detection of one of the steady states (SLN, NASP, PSTUT, or PSWB). The classification algorithm was initially piloted in Microsoft Excel (Visual Basic) using Solver and the Data Analysis Toolbox (*F*-test and *t*-test) to perform piecewise linear fitting and statistical tests. The program was then re-implemented in the platform-independent Java programming language using the Apache Commons Mathematics Library (commons.apache.org/proper/commons-math). The source code and executable of Java implementation is available at github.com/Hippocampome-Org/NeuroSpikePatterns. Also, we attached a pseudocode that allows one easily to re-implement the program in another language/environment.

**
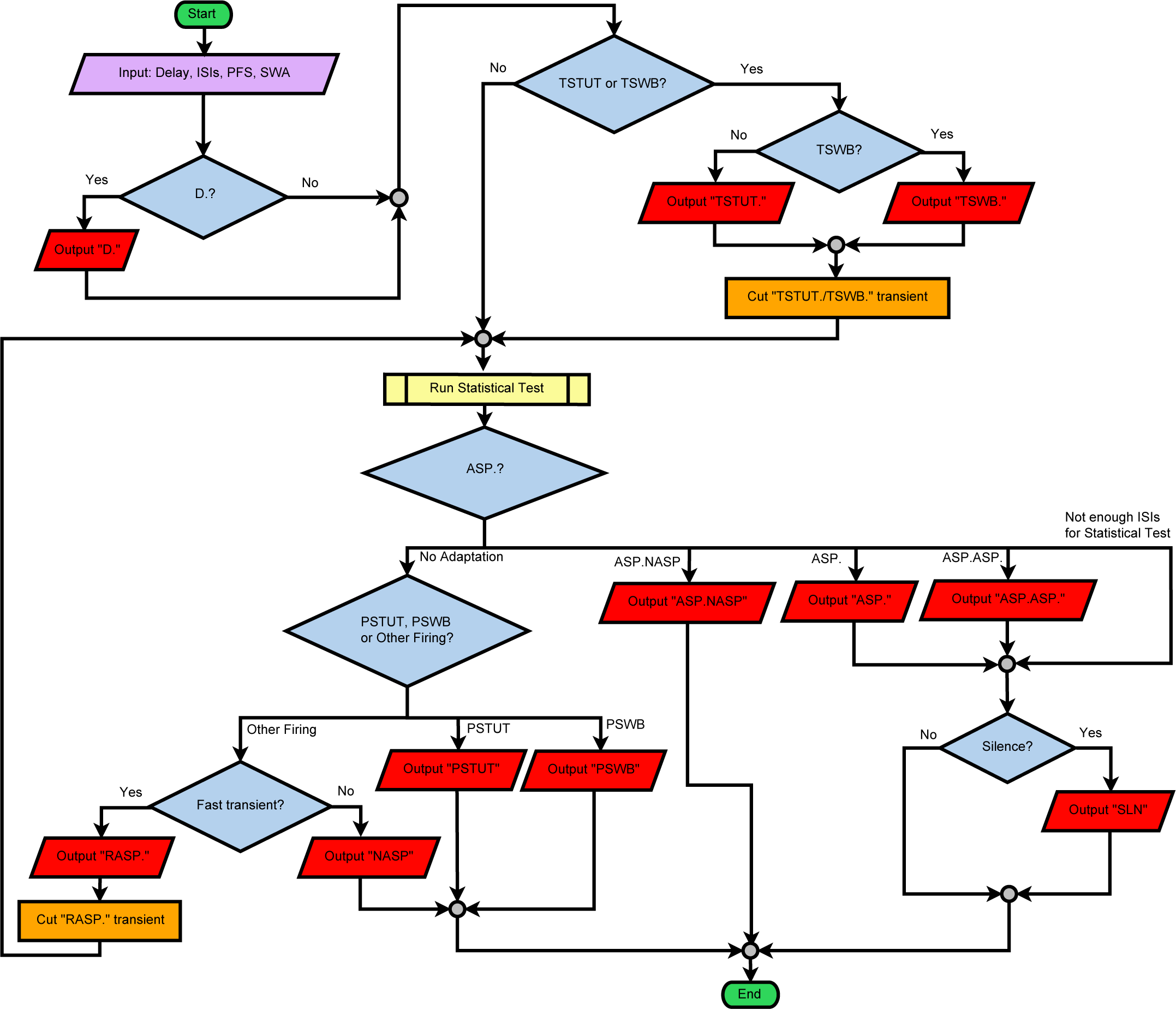
**

**Supplementary Figure S2. Flow chart of general procedure for firing pattern identification.**

See text for abbreviations.

**Implementation of the Web portal**

The implementation of Hippocampome.org supports the model-view-control software design. The model component defines the database interface and is provided solely by server-side code. The view component rendering the web pages and the control code implementing the decision logic are both served up by the server, but are run in the user’s browser. The underlying relational database ensures flexibility in establishing relations between data records.

Hippocampome.org is deployed on a CentOS 5.11 server running Apache 2.2.22 and runs on current versions of several web browsers (Mozilla Firefox, Google Chrome, Apple Safari, and Microsoft Internet Explorer). Knowledge base content is served up to the PHP 5.3.27 website from a MySQL 5.1.73 database. Django 1.7.1 and Python 3.4.2 provide database ingest capability of comma separated value annotation files derived from human-interpreted peer-reviewed literature. Hippocampome.org code is available at github.com/Hippocampome-Org.

**Supplementary Results**

**Completed firing patterns in hippocampal and other neurons**

Our classification of firing pattern elements implies the possibility of three completed single-element firing patterns (NASP, PSTUT, PSWB) and 19 completed double-element firing patterns consisting of one of four steady states (SLN, NASP, PSTUT, PSWB) preceded by one of five transients (D, ASP, RASP, TSTUT, TSWB), with exclusion of the “empty” combination D.SLN. Also, four double-transients are possible after an initial delay, resulting in an additional 16 triple-element firing patterns. Only 15 of these possible 38 completed firing patterns were discovered in literature data for morphologically identified hippocampal neuron types (Supplementary Table S1). Three additional firing patterns were found in other neurons: D.PSWB has been shown in the cultured *rutabaga* mutant giant neuron of *Drosophila*^101^*,* D.ASP.SLN in the neuron of the external lateral subnucleus of the lateral parabrachial nucleus^102^, and D.TSTUT.SLN in the striatal fast-spiking neuron^103^. We deemed 16 firing patterns as “not found but possible” (white shading and black text in Supplementary Table S3) and 4 firing patterns as “improbable” (white shading and gray text). In particular, we consider combination of stuttering and slow-wave bursting (TSWB.PSTUT or TSTUT.PSWB) as unlikely to occur under physiological conditions from a dynamical viewpoint due to incompatible underlying mechanisms. Slow-wave bursting is provided by a slow negative feedback which terminates the burst of action potential evoking slow AHP. Such feedback could be produced by different ionic mechanisms, but it is most typically based on intracellular Ca^2+^ dynamics and Ca^2+^-activated K^+^ current^31,104^ or muscarinic-sensitive K^+^ current^31^. Slow-wave bursting could be “square-wave bursting”, with one slow process, or “parabolic bursting”, with two (positive and negative feedback) slow processes^105^. In contrast, stuttering activity is associated with “elliptic bursting”^4^, where the silent phase is characterized by dumping and growing fast (spiking) oscillations as the trajectory slowly drift through bifurcation of the fast subsystem^105^. Suggested mechanism for stuttering in fast spiking interneurons includes Na^+^ “window” current that induces high frequency tonic firing, and slowly inactivating K^+^ current through KV1 channels^106^.

**Supplementary Table S3. Occurrences of completed firing patterns in hippocampal and other neurons**

|  | |  |  | **Steady States** | | | |
| --- | --- | --- | --- | --- | --- | --- | --- |
|  | |  |  | **NASP** | **PSTUT** | **PSWB** | **SLN** |
|  | **-** | | **-** | **NASP**  DG HICAP | **PSTUT**  CA1 Neurogliaform | **PSWB**  CA3 Pyramidal | **—** |
| **Transients** | **-** | | **ASP** | **ASP.NASP**  CA3 Basket-CCK | **ASP.PSTUT** | **ASP.PSWB** | **ASP.SLN**  EC MEC LV Pyramidal |
|  | **-** | | **RASP** | **RASP.NASP**  EC LV Deep Pyramidal | **RASP.PSTUT** | **RASP.PSWB** | **RASP.SLN**  CA1 Radiatum Giant |
|  | **-** | | **TSTUT** | **TSTUT.NASP**  EC LV Deep Pyramidal | **TSTUT.PSTUT** | **TSTUT.PSWB** | **TSTUT.SLN**  CA2 SP-SR |
|  | **-** | | **TSWB** | **TSWB.NASP**  CA1 Pyramidal | **TSWB.PSTUT** | **TSWB.PSWB** | **TSWB.SLN**  CA3 Pyramidal |
|  | **D** | | **-** | **D.NASP**  DG Neurogliaform | **D.PSTUT**  CA1 Bistratified | **D.PSWB**  *Drosophila* giant | **—** |
|  | **D** | | **ASP** | **D.ASP.NASP** | **D.ASP.PSTUT** | **D.ASP.PSWB** | **D.ASP.SLN**  Lat. parabrachial nucl. |
|  | **D** | | **RASP** | **D.RASP.NASP**  CA3 LMR-Targeting | **D.RASP.PSTUT** | **D.RASP.PSWB** | **D.RASP.SLN** |
|  | **D** | | **TSTUT** | **D.TSTUT.NASP** | **D.TSTUT.PSTUT** | **D.TSTUT.PSWB** | **D.TSTUT.SLN**  Striatal fast-spiking |
|  | **D** | | **TSWB** | **D.TSWB.NASP**  CA1 Axo-Axonic | **D.TSWB.PSTUT** | **D.TSWB.PSWB** | **D.TSWB.SLN** |

| **NASP** | observed in hippocampal neurons | **TSWB.PSTUT** | improbable |
| --- | --- | --- | --- |
| **D.PSWB** | observed in other neurons | **—** | impossible (no firing) |
| **D.ASP.NASP** | not found but possible |  |  |

**NASP** – HICAP (Mott *et al.*^39^, Fig. 11A); **PSTUT** - CA1 Neurogliaform (Fuentealba *et al.*^29^, Fig.5B); **PSWB** - CA3 Pyramidal (Bilkey and Schwartzkroin^32^, Fig. 1a); **ASP.NASP** - CA3 Basket-CCK (Gulyás *et al.*^107^, Fig. 1b, right); **ASP.SLN** – EC MEC LV Pyramidal (Canto and Witter^14^, Fig.10C7); **RASP.NASP** – EC LV Deep Pyramidal (Hamam *et al.*^27^, Fig.3C); **RASP.SLN** – CA1 Radiatum Giant (**Bullis et al.^108^,** Fig.5A); **TSTUT.NASP** - EC LV Deep Pyramidal (Hamam *et al.*^48^, Fig.5E); **TSTUT.PSTUT** - CA1 (Price *et al.*^30^, Fig.3A2); **TSUT.SLN** – CA2 SP-SR (Mercer et al.^97^, Fig. 3A); **TSWB.NASP** - CA1 Pyramidal (Zemankovics *et al.*^43^ , Fig. 1B); **TSWB.SLN** - CA3 Pyramidal (Hemond *et al.*^15^, Fig. 4); **D.NASP** – DG Neurogliaform (Armstrong *et al.*^44^, Fig.3A, top trace); **D.PSTUT** - CA2 Basket (Mercer *et al.*^17^, Fig. 5B)**;** **D.PSWB -** cultured *rutabaga* mutant giant neuron of  *Drosophila* (Zhao and Wu^101^, Fig.7, top left); **D.ASP.SLN -** neuron in external lateral subnucleus of lateral parabrachial nucleus (Hayward and Felder^102^, Fig.3A, top); **D.RASP.NASP** - CA3 LMR-Targeting (Ascoli *et al.*^109^, Fig. 1A); **D.TSUT.SLN** - striatal fast-spiking neuron (Sciamanna and Wilson^103^, Fig. 1C); **D.TSWB.NASP** - CA1 Axo-Axonic (Buhl *et al.*^45^, Fig. 5D). **Abbreviations:** Lat. – lateral; nucl. – nucleus.

**Supplementary References**

99. Ali, A.B. & Thomson, A.M. Facilitating pyramid to horizontal oriens-alveus interneurone inputs: dual intracellular recordings in slices of rat hippocampus. *J. Physiol.* **507**, 185-199 (1998).

100. Lacaille, J.C., Mueller, A.L., Kunkel, D.D. & Schwartzkroin, P.A. Local circuit interactions between oriens/alveus interneurons and CA1 pyramidal cells in hippocampal slices: electrophysiology and morphology. *J. Neurosci.* **7**, 1979-1993 (1987).

101. Zhao, M.L. & Wu, C.F. Alterations in frequency coding and activity dependence of excitability in cultured neurons of *Drosophila* memory mutants. *J. Neurosci.* **17**, 2187-2199 (1997).

102. Hayward, L.F. & Felder, R.B. Electrophysiological properties of rat lateral parabrachial neurons in vitro. *Am. J. Physiol.* **276**, R696-R706 (1999).

103. Sciamanna, G. & Wilson, C.J. The ionic mechanism of gamma resonance in rat striatal fast-spiking neurons. *J. Neurophysiol.* **106**, 2936-2949 (2011).

104.Xu, J. & Clancy, C.E. Ionic mechanisms of endogenous bursting in CA3 hippocampal pyramidal neurons: a model study. *PLoS One* **3**, e2056; 10.1371/journal.pone.0002056 (2008).

105. Rinzel, J. & Ermentrout, G.B. Analysis of neural excitability and oscillations in *Methods in Neuronal Modeling: From Ions to Networks* (eds Koch, C. & Segev, I.) 251–291 (MIT Press, 1998).

106. Golomb, D. *et al.* Mechanisms of firing patterns in fast-spiking cortical interneurons. *PLoS Comput. Biol.* **3**, e156; 10.1371/journal.pcbi.0030156 (2007).

107. Gulyás, A.I. *et al.* Parvalbumin-containing fast-spiking basket cells generate the field potential oscillations induced by cholinergic receptor activation in the hippocampus. *J. Neurosci.* **30**, 15134-15145 (2010).

108. Bullis, J.B., Jones, T.D. & Poolos, N.P. Reversed somatodendritic *I(h)* gradient in a class of rat hippocampal neurons with pyramidal morphology. *J. Physiol.* **579**, 431-443 (2007).

109. Ascoli, G.A. *et al.* Quantitative morphometry of electrophysiologically identified CA3b interneurons reveals robust local geometry and distinct cell classes. *J. Comp. Neurol.* **515**, 677-695 (2009).
